# Ten principles for generating accessible and useable COVID‐19 environmental science and a fit‐for‐purpose evidence base

**DOI:** 10.1002/2688-8319.12041

**Published:** 2021-02-04

**Authors:** Andrew N. Kadykalo, Neal R. Haddaway, Trina Rytwinski, Steven J. Cooke

**Affiliations:** ^1^ Canadian Centre for Evidence‐Based Conservation Department of Biology and Institute of Environmental and Interdisciplinary Science Carleton University Ottawa Canada; ^2^ Mercator Research Institute on Global Commons and Climate Change Berlin Germany; ^3^ Stockholm Environment Institute Stockholm Sweden; ^4^ Africa Centre for Evidence University of Johannesburg Johannesburg South Africa

**Keywords:** conservation evidence, COVID‐19, data hygiene, environmental evidence, evidence synthesis, evidence‐based conservation, evidence‐informed decision‐making, knowledge co‐production, open science, registered reports

## Abstract

1. The ‘anthropause’, a period of unusually reduced human activity and mobility due to COVID‐19 restrictions, has serendipitously opened up unique opportunities for research on how human activities impact the environment.

2. In the field of health, COVID‐19 research has led to concerns about the quality of research papers and the underlying research and publication processes due to accelerated peer review and publication schedules, increases in pre‐prints and retractions.

3. In the field of environmental science, framing the pandemic and associated global lockdowns as an unplanned global human confinement experiment with urgency should raise the same concerns about the rigorousness and integrity of the scientific process. Furthermore, the recognition of an ‘infodemic’, an unprecedented explosion of research, risks research waste and duplication of effort, although how information is used is as important as the quality of evidence. This highlights the need for an evidence base that is easy to find and use – that is discoverable, curated, synthesizable, synthesized.

4. We put forward a list of 10 key principles to support the establishment of a reproducible, replicable, robust, rigorous, timely and synthesizable COVID‐19 environmental evidence base that avoids research waste and is resilient to the pressures to publish urgently. These principles focus on engaging relevant actors (e.g. local communities, rightsholders) in research design and production, statistical power, collaborations, evidence synthesis, research registries and protocols, open science and transparency, data hygiene (cleanliness) and integrity, peer review transparency, standardized keywords and controlled vocabularies.

## Graphical abstract

Ten Key Principles for a Robust COVID‐19 Environmental Evidence Base that Avoids Research Waste and is Resilient to the Pressures to Publish Urgently.

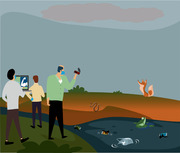

## 1 INTRODUCTION

COVID‐19, the novel Coronavirus infectious disease of 2019, has resulted in an ongoing pandemic impacting all facets of human society. This includes impacts to environmental science, policy and practice. The pandemic has dramatically reduced human activity and movement globally. At its peak, approximately half of the human population was strongly encouraged to stay in their homes to reduce the virus spread (Bates, Primack, Moraga, & Duarte, 2020). This period of unusually reduced human activity and mobility has been recently termed the ‘anthropause’ (Rutz et al., 2020). While the impacts of the virus (and associated ‘lockdowns’) to human well‐being and welfare (including to environmental researchers and practitioners) are overwhelmingly negative, the pandemic serendipitously opens up unforeseen and unique opportunities for research during and after the outbreak. Namely, the anthropause represents an unprecedented, once‐in‐a‐lifetime opportunity to investigate how human activities impact ecosystem functions and processes, wildlife and other biodiversity across the full range of ecosystems (Bates et al., 2020; Primack et al., 2020).

While research on COVID‐19 in environmental science is framed as opportunistic, in the field of health, where COVID‐19 research is clearly duty‐bound, the pandemic has led to one of the biggest explosions of scientific literature on record. For example, in May 2020 more than 4000 papers were published on the novel coronavirus in a single week alone and the COVID‐19–related scientific literature is estimated to be doubling every 20 days (Brainard, 2020); we are not only in a pandemic but also an *infodemic* (Zarocostas, 2020). This explosion has led to concerns about the quality of research papers and the underlying research and publication processes (i.e. questionable research practices). Some have called this an era of ‘panicky, pandemic publishing’ (Caulfield, Bubela, Kimmelman, & Ravitsky, 2020) in which this deluge of research is arguably of poor quality and prepared in a rushed manner sabotaging a potentially effective evidence base (e.g. Glasziou, Sanders, & Hoffmann, 2020; Rzymski et al., 2020). Some medical publishers have accelerated peer review and publication schedules. For example, 14 medical journals publishing COVID‐19 content have halved the average time from submission to publication to approximately 60 days (Brainard, 2020; Kwon, 2020). Another estimate found an astounding acceleration in publication speed with a decrease in the time from submission to acceptance of COVID‐19 journal articles from a median of 100 days to just 6 days (Palayew et al., 2020). Some of the concern about quality also relates to the concept of ‘preprint surge’ (Kwon, 2020) – a rise in preprints which are not peer‐reviewed in a bid to make research findings publicly available more quickly. For example, as of May 2020, 32% of COVID‐19 papers on the National Institute of Health's COVID‐19 portfolio are preprints (ASAPbio, 2020) while Fraser et al. (2020) found that COVID‐19 preprints are accessed and distributed by academic, public and news media at least 15 times more than non‐COVID‐19 preprints. Further, in the field of health, retractions of high‐profile coronavirus studies (Ledford & Van Noorden, 2020) have led to a corresponding surge in retractions and a ‘retraction watch’ of COVID‐19 papers (https://retractionwatch.com – as of November, 2020 at 39 retracted COVID‐19 studies). While robust evidence does not necessarily depend on the speed in which it is generated (see Roche et al., 2019), faster review and editorial processes and preprint surge raise concerns about whether they come at the expense of research quality and integrity (i.e. care, rigour, robustness, transparency, reproducibility, replicability, accessibility).

Furthermore, this flood of health evidence has resulted in a high risk of research waste (see Glasziou et al., 2020) because more researchers are working on related topics; research is often conducted concurrently without pre‐registration; opportunities for establishment of collaborations could be sidelined in favour of rapid completion; and, research needs are global in nature and are conducted by, as yet, disconnected research communities. As a result of this explosion of evidence, researchers in health have developed a suite of platforms and services to curate and catalogue evidence on COVID‐19 (e.g. COVID‐END, https://www.mcmasterforum.org/networks/covid‐end; evidence aid, https://evidenceaid.org/evidence/coronavirus‐covid‐19/): but these all require considerable efforts to overcome a fragmented evidence base that is difficult to discover and synthesize.

We are already seeing the establishment of an evidence base (e.g. Everard, Johnston, Santillo, & Staddon, 2020; Manenti et al., 2020; Rupani et al., 2020; Zambrano‐Monserrate, Ruano, & Sanchez‐Alcalde, 2020) on the environmental impacts of the COVID‐induced anthropause, albeit at a slower pace. Calls in the environmental community are encouraging research and communication to avoid ‘missed opportunities’ (see Bates et al., 2020; Corlett et al., 2020; Evans et al., 2020; Primack et al., 2020; Rutz et al., 2020). This is evidenced by the framing of the pandemic and associated global lockdowns as an unplanned ‘Global Human Confinement Experiment’ to investigate human impacts on the environment – from biodiversity to ecosystems to protected areas (Bates et al., 2020; Primack et al., 2020). Open calls for COVID‐19–related papers also demonstrate this urgency in opportunity (e.g. special issue in the journal *Biological Conservation* on ‘Covid‐19 and Conservation’, *Frontier*’s special collection ‘Assessment of the Impact of Covid‐19 Pandemic on Water, Environment and Related Ecological and Human Systems).

Environmental research into the impacts of the pandemic will require careful planning to avoid issues of biased and questionable research and data quality. Expediting research and communication increases the risk that research and its underlying data are fragmented, inaccurate or biased or of poor quality or low power (Brainard, 2020; Ledford & Van Noorden, 2020). It also increases potential research waste: the risk that research could be redundant, unnecessary or misleading (Glasziou et al., 2020) which was a concern in environmental science even prior to the pandemic (Buxton et al., 2020). To avoid such issues, researchers and funding agencies must proceed with caution to ensure their science is reproducible, replicable, robust, synthesizable and ultimately impactful and useable. We need to acknowledge that in environmental science most of this work was not planned and therefore comes with trade‐offs between rigour and timeliness. Due to its unplanned nature and sense of urgency, this will likely also precipitate conditions for research carelessness such as a lack of replication or appropriate experimental controls.

## 2 TEN KEY PRINCIPLES FOR A ROBUST COVID‐19 ENVIRONMENTAL EVIDENCE BASE

We offer a list of 10 key principles to support the establishment of a robust COVID‐19 environmental evidence base. These key principles are of course important considerations in any scientific enterprise, but we emphasize them here as a special reminder given the current context of the pandemic. These considerations are especially important given potential accelerations to environmental science research and publication processes and the possible negative implications to research quality and integrity.

**1**. *Engage all relevant actors (rightsholders, stakeholders, practitioners, policy makers, partners, etc.) in research design and production*. We acknowledge that co‐design and co‐production of research is inherently difficult, risk‐laden and costly (Sutherland, Shackelford, & Rose, 2017; Oliver, Kothari, & Mays, 2019). However, where time and financial resources allow, engage relevant actors in long‐term partnerships where knowledge could be co‐produced, or at least co‐assessed (Sutherland et al., 2017). This will help ensure the generated science is integrated, appropriate, relevant, useable and ultimately effective and implemented. Such engagement could include, for example developing management strategies, informing literature searches and study designs. Institutions must of course be willing to provide the time and resource requirements to make such engagement possible and mainstream.

**2**. *Conduct power analyses and avoid underpowered research. To offset small and short‐term studies, pursue collaborations that permit larger sample sizes*. Many studies, especially in the environmental sciences, are still underpowered, despite Jacob Cohen's warning in 1962 of the issues of conducting studies with insufficient statistical power to detect effect sizes and interaction effects (Parker et al., 2016; Smaldino & McElreath, 2016). In general, the larger the sample size, the greater the power, which increases the precision of estimates. The greater the sample size, the less it will also be affected by sampling error. While larger sample sizes cost more money and time, if sample sizes are small, the power of any statistical test will usually be low, and the conclusion reached – meaningless. In other words, powerful tests can detect small differences, weak tests can only detect large differences. Low power results in more false negatives in which a true effect was indeed there, but it was not detected in the study. Funders should therefore make power analyses a conditional requirement for award applications. Note, however, that the sample size or statistical power is inconsequential if sampling is non‐representative or non‐random (i.e. biased). Many of our sampling units (whether organism, population or ecosystem) are dynamic and subject to flux. This makes observation and experimentation especially complex and messy. Collaborative projects with standardized methods across geo‐spatial scales would permit larger sample sizes and stronger confidence in the evidence collected even in shorter‐term studies. For example, Canada's ‘Experimental Lakes Area’ and its large number of replicates (58 formerly pristine lakes) allow for long‐term, whole‐lake investigations and is famously credited for providing the most compelling evidence for phosphorus being the cause of anthropogenic eutrophication. The Many Labs project (see Klein et al., 2018) presents an approach to replicating previously completed experiments, but the infrastructure is applicable to any research question, including novel ones in environmental sciences, and allows for large‐scale study designs with spatial replication across different contexts. Protocols from Many Labs standardize and carefully plan replication that permit huge increases in sample sizes resulting in analysis of variables and replication not possible in a single case study (e.g. ‘Each protocol was administered to approximately half of 125 samples that comprised 15,305 participants from 36 countries and territories’.). While COVID‐19 research was likely initially reactive, now with second waves and associated follow‐up lockdowns we have opportunities to be more proactive with the design of research to make them more powerful for detecting effects. We call for collaborative and additive thesis projects to avoid piecemeal publications. Collaboration, where and when possible, is therefore a potential solution to increase sample size and statistical power. It would increase research quality opposing ‘publish or perish’ incentives.

**3** *. In policy, practice, regulatory or statutory contexts, pursue syntheses of primary studies, which have a greater* a priori *inferential strength; use them to analyse different novel factors (variables) associated with variation among studies in effect size that could not be analysed in single studies; and provide a more robust evidence base than single studies*. Syntheses of primary studies (systematic reviews and meta‐analyses) have a greater inferential strength to primary studies by reducing the potential for bias by transparently selecting studies, helping to resolve (or at least make sense of) conflicting studies, increase sample sizes testing a particular question or hypothesis, are replicable (in principle) and provide a reliable basis for decision making that avoids ‘cherry‐picking’. Evidence synthesis reduces research waste and duplication of effort by making use of existing evidence. Thus, the results of several primary studies combined in a systematic review to provide an overarching view of the topic will potentially have much greater inferential power and greater and broader ability to examine patterns than any individual primary study. Evidence synthesis will therefore improve the quality of the evidence base. Consequently, where more than one study exists for a given environmental science question, policy and practice should be influenced by the evidence hierarchy and pyramid – where at the top of the hierarchy/pyramid are systematic reviews and meta‐analyses which are ‘higher levels of evidence’ than single studies (Glover, Izzo, Odato, & Wang, 2006) which should feed into guidance for policy and practice (Dicks, Walsh, & Sutherland, 2014).

In environmental science, evidence synthesis approaches can be applied to a multitude of diverse research questions, for example, the impacts of reindeer/caribou (*Rangifer tarandus L*.) on Arctic and alpine vegetation (Bernes, Bråthen, Forbes, Speed, & Moen, 2015), the effectiveness of road mitigation in reducing road‐kill (Rytwinski et al., 2016) or the flood control services of wetlands (Kadykalo & Findlay, 2016). COVID‐19–relevant research could also be addressed using evidence synthesis, for example what are the impacts of lockdown‐induced reduced human personal vehicle traffic on collisions with wildlife? Evidence syntheses are also a valuable alternative to primary research during COVID‐19 at a period when researchers are impacted by the lack of key resources and access to research laboratories or field sites – permitting researchers to continue contributing to evidence‐based environmental policy and practice.

Thus, evidence syntheses are a powerful means of collating and learning from rapidly expanding bodies of evidence, such as original COVID‐19–related research. Once COVID‐19 research becomes published and available (i.e. synthesis ready), evidence syntheses should be pursued to maximize the inference and value of the collected evidence.

**4**. *Adopt research registries*. Publication bias (when the outcome of a research study influences the decision on whether to publish it – ‘the file drawer problem’) and selective reporting bias (systematic differences between reported and unreported findings) can lead to an overestimation of treatment effects and affects the validity, replication and transparency of research (e.g. Fanelli, 2010). However, research that is difficult to publish in a peer‐reviewed format due to non‐significant, negative or a lack of novel results still has tremendous value and should be considered within the entire evidence base. Research registries, an idea borrowed from clinical trial registries in the field of health, require researchers to register prior to undertaking research leaving a digital ‘trail’ for research studies; see Parker, Fraser, and Nakagawa (2019), which call for more pre‐registration and registered reports (next principle) in conservation science. Trial registration systems document titles, summaries and author affiliations/funding information in a database to combat publication bias and selective reporting. Such registries would also aid in identifying opportunities to reduce redundancy or enhance replication which builds strength of evidence. For example, there are over 1300 registered trial registries for randomized clinical trials investigating COVID‐19 medical prevention and treatment which allow people to identify studies that were initiated (see Karlsen et al., 2020). The methods of these studies can then be critiqued irrespective of the final findings or terminal publication destination, helping to mitigate publication bias.

**5**. *Adopt and publish freely accessible research protocols*. Selective reporting of findings (i.e. selective reporting bias), confirmation bias (i.e. preferential treatment of observations which align with one's beliefs), statistical manipulation (e.g. *p*‐hacking) and HARKing are common issues in scientific research (Forstmeier, Wagenmakers, & Parker, 2017; Head, Holman, Lanfear, Kahn, & Jennions, 2015; Murphy & Aguinis, 2019; Parker et al., 2016; Saini et al., 2014). A research protocol sets out the plans for the conduct of research and is integral in producing research that is robust against *post hoc* changes in methods and scope (also known as *mission creep*; Haddaway et al., 2020). Authors can also use a public protocol to solicit feedback on research design through peer review before the research commences. Registered reports are prepared by researchers that detail all of the study protocols; anticipating as many of the potential issues that might arise with the data and study as possible and detailing what will be done in each case (e.g. how outliers are to be handled, when sufficient sample size has been reached and data collection can stop). Reports are time stamped on submission and serve as an official record that can be referred to. They provide reviewers with greater confidence in the results. Pre‐registered reports can be peer‐reviewed, and registrations can be embargoed for a later date if the information contained within the report is confidential or sensitive to ‘scooping’. The Centre of Open Science's Open Science Framework and certain journals (e.g. *Conservation Biology*, *Ecology and Evolution*, *Ecological Solutions and Evidence*, *Environmental Evidence*, *Royal Society Open Science*) manage registries or facilitate registered manuscripts. This can help document research progress including where and when a research study's findings are peer‐reviewed and published or where, for example, a preprint is a terminal destination (e.g. reviewers did not find the work novel, not enough funding to cover revisions). Researchers and funders should endeavour (or be required) to publish freely accessible research protocols (also referred to as *registered reports* or *pre‐registration*) *a priori*. Freely accessible research protocols help establish research that is reproducible, replicable, and rigorous safeguarding the integrity of upcoming COVID‐19–related research.

**6**. *Adopt Open Science principles to increase transparency and (re‐)use of data, methods, and papers*. Closed science – data that are not made public, study findings that are not readily and easily synthesizable, metadata that are not descriptive and understandable – risk perpetuating the reproducibility crisis (Baker, 2016) and upholding the perception of academic science as a disconnected ivory tower. Good data curation will reduce COVID‐19 research waste and help ensure that data and the associated findings can be applied and re‐used to answer broader and multiple questions and hypotheses through replication studies and meta‐analyses. When making data publicly available, researchers should conform to FAIR principles (Wilkinson et al., 2016) when possible to make data ‘F’indable, ‘A’ccessible, ‘I’nteroperable and ‘R’eadable. Moreover, reporting and archiving of data and results should be transparent, systematic and comprehensive (e.g. provide sufficient statistical information such as mean, standard deviation or some estimate of precision and sample size for the various groups). It should also include archiving of raw data, metadata, analytical scripts and calculations or transformations of the data by authors. This will help facilitate both human and machine readability and synthesizability. Moreover, it will minimize inequities, injustices and biases in COVID‐19 science such as minimal/insufficient peer review, selective reporting, editorial bias, publication bias and claims unsupported by evidence. It also benefits researchers with increases in citations, media attention, potential collaborators, job opportunities and funding opportunities (McKiernan et al., 2016).

**7**. *Implement Open Science training and Open Education*. Following from above, research institutions should implement Open Science training opportunities, although we acknowledge these benefits to the evidence base are likely not to be realized for years or decades. However, teaching of open science at post‐secondary education levels will also help to reduce research waste and help ensure that COVID‐19–related science can be applied and re‐used to answer broader and multiple questions and hypotheses. By extension, we call for any Open Science training to feature Open Education, namely, free‐to‐use educational materials.

**8**. *Take care to maintain data hygiene (cleanliness) and data integrity*. COVID‐19 research is inherently time‐sensitive. As research and publication schedules accelerate, take extra care in data cleansing and management to maintain quality of data under pressure. Carelessness could result in omitted data, duplicated data, incomplete data, mistreatment of outliers, improperly curated data, inclusions of inappropriate data, etc. For (recent) example, (i) the UK government's contact tracing programme fiasco, scrambling to reach up to 50,000 people because 15,841 positive COVID‐19 results were omitted due to a ‘catastrophic’ Microsoft Excel data error (Halliday, Walker, & Campbell, 2020); (ii) a health study on COVID‐19 (Logunov et al., 2020) has come under scrutiny (Andreev et al., 2020) for data inconsistencies, perhaps duplicated data; (iii) an analysis of whether male principal investigators (PIs) publish with women in ecology and zoology was criticized for mistreatment of data, including single author papers and for including last authors (traditionally PIs) in the category of ‘proportion of female co‐authors’ (Salerno, Páez‐Vacas, Guayasamin, & Stynoski, 2020). In sum, when under pressure to publish COVID‐19–related research urgently be vigilant about data quality – annotate your work, record meta‐data, be a detective about data and back up your data.

**9**. *Increase* peer review *transparency*. A rise in preprints and accelerated peer review and publication schedules – a concern of COVID‐19–related research (Fraser et al., 2020; Kwon, 2020; Palayew et al., 2020; Rzymski et al., 2020; Teixeira da Silva, 2020) – risks reducing the quantity and quality of peer reviews. Journals should take steps to be transparent about peer reviews of poor quality and the length of peer reviews; see Parker et al. (2018) for an excellent checklist of important questions to ask to improve transparency with respect to the rigour of study design and analyses and the presentation of the methods and results. We also call for the increased use of open peer review – that is, open review reports, and/or open participation in the review process from the wider community. Post‐publication peer review is one such example in which manuscripts are checked by the editor to ensure it is appropriate and meets the criteria and requirements of the journal. Succeeding, the article is published online, and the peer review process including editors, reviewers and the broader community begins openly and transparently; aiming to avoid editorial bias while increasing the speed of publication.

**10**. *Establish standardized keywords and controlled vocabularies for COVID‐19–relevant and related environmental research*. Problems with ‘research discovery’ (i.e. a lack of easily accessible and searchable research) is a well‐established barrier to research use. This has been demonstrated by COVID‐19 ‘vocabulary chaos’ in the medical realm, emphasizing the need for controlled vocabularies and living search strategies (Shokraneh and Russell, 2020) which environmental science can learn from. A lack of efficient research discovery is limited by researchers who do not [know how to] make research discoverable, a dearth of training in research discovery, and research cataloguing systems that are not fit‐for‐purpose. Information overload, heterogeneous terminology, a lack of training in research discovery, and research in multiple languages contribute to discoverability challenges. Powerful keywording like ‘subject headings’ (e.g. MeSH) – peer‐reviewed words or phrases in a database to describe a certain concept – can increase searchability, accessibility and use of research. For an environmental research example, Conservation Evidence (https://www.conservationevidence.com) has made great efforts to build ontologies for their 2399 (as of November 2020) conservation actions or interventions to conserve wildlife and ecosystems. Standardized keywords and subject headings if assigned and hyperlinked effectively by bibliographic databases can facilitate access to the entire evidence base on a particular subject (e.g. COVID‐19 and biological conservation). Thus, building on established ontologies in titles, abstracts and especially keywords will aid research discovery. Established keywords and controlled vocabularies would also benefit researchers, who generally lack research discovery training, in effectively ascribing their own research to relevant and accessible titles, abstracts and keywords.

## 3 CONCLUSION

We call on the entire research community to be proactive in abiding by these 10 principles to generate transparent, accessible, replicable, equitable, inclusive and rigorous science in the face of pressures for urgently publishing research on the ‘Global Human Confinement Experiment’. We realize that many of these principles demand more time from researchers, publishers and funders. However, we emphasize that taking these precautions and steps prior to research, analysis and publication will have a large payoff. These principles also work towards prioritizing high‐quality research methods over the quantity of (peer‐reviewed) publications. There will be other important and specific considerations for environmental research into the impacts of the pandemic not covered here. For example, researchers should beware in their treatment of time periods, as pandemic‐related impacts to the environment (and policy and human compliance responses) have not been uniform. This likely requires breaking down the ‘anthropause’ into logical sub‐periods. Ultimately, together, these principles will minimize questionable research practices and make COVID‐19 research more impactful, accessible and (re‐)useable by enabling access to evidence and the conditions for evidence‐informed decision making (Salafsky et al., 2019).

## AUTHORS’ CONTRIBUTIONS

NRH and SJC conceived the original idea. ANK wrote the manuscript. All authors provided critical feedback and helped shape the ideas and the manuscript. All authors gave final approval for publication.

### PEER REVIEW

The peer review history for this article is available at https://publons.com/publon/10.1002/2688-8319.12041.

## Data Availability

This manuscript does not use data; therefore, no data are archived.
